# “Decoding” Angiogenesis: New Facets Controlling Endothelial Cell Behavior

**DOI:** 10.3389/fphys.2016.00306

**Published:** 2016-07-21

**Authors:** Raj Sewduth, Massimo M. Santoro

**Affiliations:** ^1^Laboratory of Endothelial Molecular Biology, Department of Oncology, Vesalius Research Center, VIB, KU LeuvenLeuven, Belgium; ^2^Department of Molecular Biotechnology and Health Sciences, University of TurinTorino, Italy

**Keywords:** angiogenesis, redox signaling, Notch signaling pathway, flow dynamics, metabolism

## Abstract

Angiogenesis, the formation of new blood vessels, is a unique and crucial biological process occurring during both development and adulthood. A better understanding of the mechanisms that regulates such process is mandatory to intervene in pathophysiological conditions. Here we highlight some recent argument on new players that are critical in endothelial cells, by summarizing novel discoveries that regulate notorious vascular pathways such as Vascular Endothelial Growth Factor (VEGF), Notch and Planar Cell Polarity (PCP), and by discussing more recent findings that put metabolism, redox signaling and hemodynamic forces as novel unforeseen facets in angiogenesis. These new aspects, that critically regulate angiogenesis and vascular homeostasis in health and diseased, represent unforeseen new ground to develop anti-angiogenic therapies.

## New aspect of old player in angiogenesis: an update

Angiogenesis is defined as the process of sprouting new blood vessels from preexisting vasculature. Neo-vessel formation is required for many physiological processes, such as embryogenesis, cardiovascular maturation, tissue repair and regeneration (Schmidt and Carmeliet, 2010). Angiogenic processes need to be finely balanced during development and adulthood, because excessive or insufficient angiogenesis contributes to pathologies, ranging from cancer, macular degeneration, and retinopathy to impaired repair of ischemic tissues (Carmeliet, 2005).

The main driver of angiogenesis is the arrangement of endothelial cells (EC) in tip and stalk cells. Tip cells form filopodia that invade surrounding tissue, leading the path of neo-vessel formation (Eilken and Adams, 2010). This was described in the mouse retina and in the zebrafish intersegmental vessels. Vascular Endothelial Growth Factor (VEGF) and Notch signaling pathways are vital for tip cell differentiation (Adams and Alitalo, 2007). VEGF signaling promotes angiogenesis through the secreted VEGF factor; while membrane bound Notch protein is cleaved upon stimulation to modify gene expression during neuronal and cardiovascular development (Gianni-Barrera et al., 2011).

**Figure 1:**
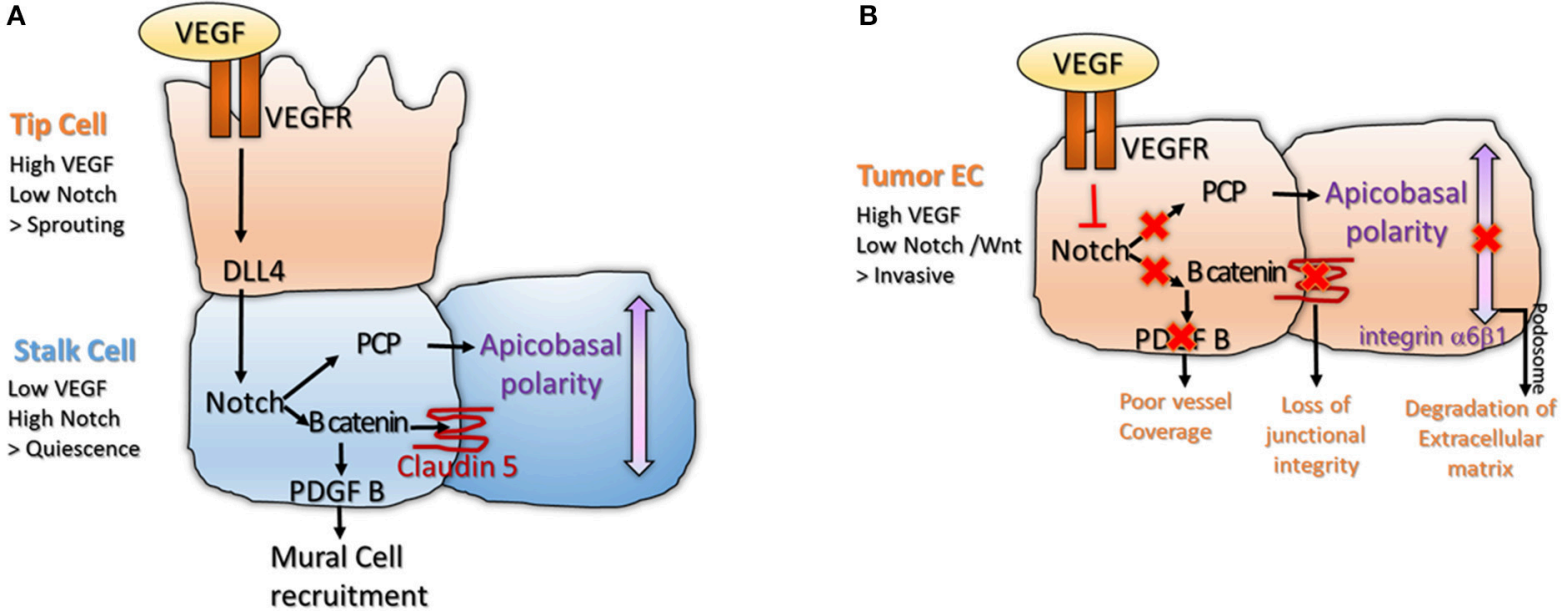
**(A)** Interplay between VEGF and Notch signaling is important for tip and stalk cell differentiation in physiological angiogenesis. The tip cell is characterized by high VEGF and low Notch levels, inducing sprouting. The stalk cell expresses low VEGF and high Notch, leading to activation of B catenin that stabilizes junctions and enables mural cell recruitment (Gavard and Gutkind, 2008; Reis et al., 2012). High Notch signaling also enables polarization of the stalk cell, a process important for lumenization (Phng et al., 2009); **(B)** The balance between VEGF and Notch signaling is lost in tumor endothelium. High VEGF and low Notch makes the tumor endothelium invasive and unstable. Loss of Notch downregulates Wnt canonical and non-canonical pathway, disrupting junctional integrity and vessel coverage (Fan et al., 2012; Chatterjee et al., 2013). High VEGF promotes sprouting and formation of podosomes at the basal side of the cell, a structure important for degradation of the extracellular matrix and cell motility (Seano et al., 2014).

VEGF Receptor 2 and 3 (VEGFR2/R3) are expressed strongly in tip cells, leading to activation of VEGF pathway in these cells (Gerhardt et al., 2003). It has been shown recently that upon binding of the ligand to VEGFR, the receptor complex would be internalized by clathrin-mediated process of endocytosis. This would require protein of the Planar Cell Polarity (PCP) pathway, Proteinase-Activated Receptor-3 (Par3), and Atypical Protein Kinase C (aPKC) (Nakayama et al., 2013; Figure 1A). VEGF pathway promotes lamellipodia and filopodia formation, giving the sprouting phenotype to the tip cell. VEGF signaling induces expression of Delta-Like 4 (Dll4). Dll4 activates Notch signaling in the neighbor stalk cells, which down-regulates VEGFR expression, giving a non-sprouting quiescent phenotype to the stalk cell (Tammela et al., 2011; Benedito et al., 2012; Ramasamy et al., 2014; Figure 1A).

Notch is important for acquisition of the barrier function and polarity in the stalk cell. Both processes are important for lumen morphogenesis. Notch pathway interacts with the Wnt pathway leading to expression of B-catenin. B-catenin has two functions in the stalk cell. It acts as a transcription factor to induce transcription of Platelet Derived Growth Factor B (PDGF-B), leading to recruitment of mural cell (Reis et al., 2012). B-catenin acts at cell junctions where it stabilizes Claudin 5 and Zona Occludens 1 (ZO1), components of the tight junctions (Gavard and Gutkind, 2008; Figure 1A). Other components of the Wnt pathway such as Frizzled 4 are required for the maintenance of the barrier function in stalk cells (Wang et al., 2012; Zhou and Nathans, 2014). Notch signaling would also interact with the non-canonical Wnt/ PCP pathway (Phng et al., 2009), leading to apico-basal polarization of the EC (Figure 1A). Apico-basal polarity is vital for vessel lumenization; as after lumen formation, one side of the EC is in contact with the blood flow and the other side is attached to the basement membrane (Descamps et al., 2012; Sewduth et al., 2014). PCP relocates Podocalyxin (POXL) to the apical membrane, where it regulates vascular permeability and integrin-alpha5 to the basal membrane, where it participates in EC attachment to the basement membrane (Figure 1A). Interestingly, basal membrane directly promotes activation of Notch signaling in stalk cells via Laminin-alpha4 (Lama4), inhibiting tip cell formation (Stenzel et al., 2011).

Interestingly, tumor endothelium is also exposed in the tumor micro-environment to high level of VEGF-A, that down-regulates Notch. However, while in normal physiology, this balance is carefully regulated leading to formation of organized structures; in tumors, VEGF signaling is deregulated and not counterbalanced, making the tumor endothelium chaotic and unstable (Carmeliet and Jain, 2011). As loss of Wnt accompanies loss of Notch signaling, tumor ECs are unable to recruit pericytes and stabilize their junctions, leading to vascular leakage (Dudley, 2012). Strong VEGF and loss of polarity triggers formation of endothelial podosome rosettes where Integrin-alpha6beta1 accumulates. Podosomes are structures that degrade extracellular matrix, a process essential for tumor EC invasiveness (Seano et al., 2014; Figure 1B). High VEGF and low Notch levels also reorganize the actin cytoskeleton, promoting lamellipodia formation (Fan et al., 2012; Chatterjee et al., 2013; Figure 1B). In conclusion, in normal endothelium, VEGF signaling is active in the tip cell where it promotes sprouting; while Notch signaling is active in the stalk cell where it favors lumen morphogenesis and acquisition of barrier properties. In tumor endothelium, this balance is disrupted making the tumor EC invasive, fragile and leaky.

## Metabolism and redox signaling: new mechanisms driving angiogenesis

**Figure 2:**
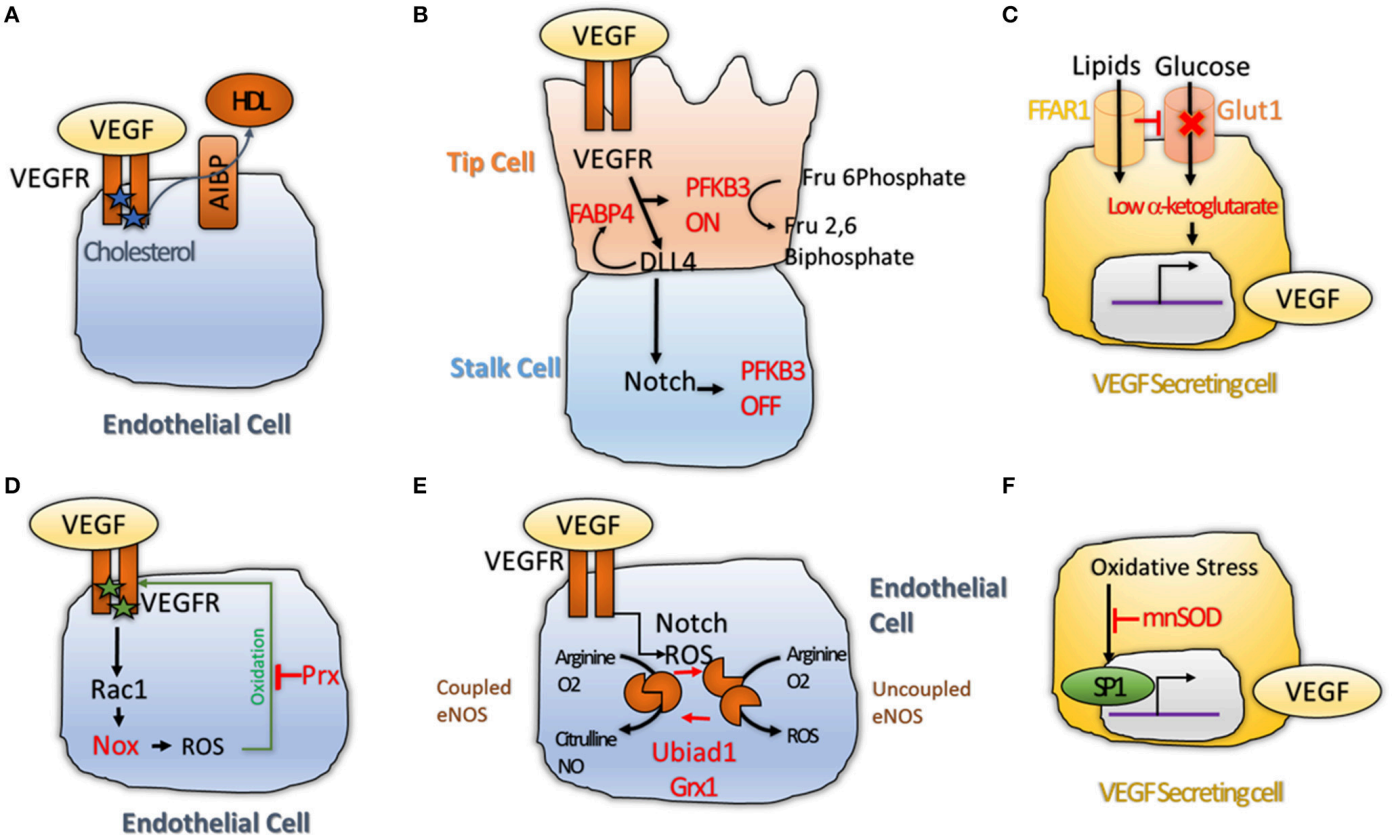
**(A)** Cholesterol maintains VEGFR2 active as dimers. Transfer of Cholesterol to HDL through the AIBP lipid transporter makes VEGFR2 inactive by inducing its monomerization (Avraham-Davidi et al., 2012); **(B)** VEGF and Notch interplay regulates Phosphofructokinase-2/Fructose-2,6-Bisphosphatase 3 (PFKFB3) activity. PFKB3 is active in tip cell, where glycolysis is promoted to favor sprouting of the tip cell. While in the stalk cell, High Notch levels shuts down PFKB3 activity, leading to quiescence (De Bock et al., 2013; Schoors et al., 2014); **(C)** Free Fatty Acid Receptor 1 (FFAR1) activates VEGF-A expression. FFAR1 is a lipid transporter that down-regulates Glut1 glucose transporter expression when free lipids are available. This reduces the levels of alpha-ketoglutarate in the cell leading to activation of VEGF-A transcription (Joyal et al., 2016); **(D)** VEGFR2 promotes ROS production via Rac1 and Nox2 (Diebold et al., 2009). High level of ROS induce oxidation of VEGF2 on two cysteine residues, making it inactive. Antioxidant enzyme Peroxiredoxin2 (Prx2) can buffer the ROS levels to keep VEGFR2 cysteines in a reduced state to protect its activity (Kang et al., 2011); **(E)** Redox balance regulates the activity of endothelial Nitric Oxide Synthase (eNOS)that can produce Nitric Oxide (NO) when in coupled conformation and generates ROS (•2^−^) when in an uncoupled conformation. Notch and ROS levels are regulators of this balance. Two antioxidant enzymes, the prenyltransferase Ubiad1 and Glutaredoxin1 (Grx1) were shown to promote shift of eNOS from uncoupled, to coupled conformation; showing that antioxidants are essential for maintenance of the NO balance and normal cell physiology (Chen et al., 2013; Mugoni et al., 2013); **(F)** ROS were shown to increase VEGF-A transcription via the transcription factor SP1 (Gonzalez-Pacheco et al., 2006); while antioxidant enzyme Manganese-dependent Superoxide Dismutase (MnSOD) was shown to block this process (Wang et al., 2005), demonstrating that redox balance regulates VEGF secretion directly.

An emerging concept is that defined metabolic pathways are vital for angiogenesis. In particular, lipid and glucose metabolism would be critical in ECs at different levels. It was shown that cholesterol at tip cell membrane is important for dimerization of VEGFR2. As VEGFR2 can bind VEGF only when it forms a dimer, Apoliprotein A-I Binding Protein (AIBP) that promotes efflux of cholesterol from the membrane to High-Density-Lipoprotein (HDL) inhibits VEGF signaling and sprouting angiogenesis (Fang et al., 2013; Figure 2A).

On the other hand, Apoliprotein-B (ApoB) that forms Low-Density-Lipoprotein (LDL) induces downregulation of VEGFR1 a decoy receptor that sequesters VEGF from VEGFR2 binding, suggesting that ApoB could activate VEGF signaling (Avraham-Davidi et al., 2012; Figure 2A). Cholesterol esterification would also impair neo-angiogenesis by reducing the amount of cholesterol available to stabilize the VEGFR2 dimers (Angius et al., 2015). The adipogenic protein Fatty-Acid-Binding-Protein 4 (FABP4) is also required for VEGFR2 downstream signaling, and is itself regulated by Dll4 (Harjes et al., 2014; Figure 2B). More recently, a new role for fatty-acid metabolism via Carnitine Palmitoyltransferase 1A (CPT1) was described (Schoors et al., 2015). Reduction of Fatty Acid Oxidation (FAO) by silencing CPT1A in ECs impaired *de novo* nucleotide synthesis for DNA replication. CPT1 blockade in mice also inhibited pathological ocular angiogenesis, showing the potential of FAO blockers to block angiogenesis (Schoors et al., 2015).

Angiogenic EC are addicted to glucose, resembling similarity with tumor cells. An interesting work describe a clear link between glucose metabolism and angiogenesis. De Bock et al. demonstrated that even if they are exposed to high oxygen concentration due to blood flow, endothelial cells rely on glycolysis and not oxidative phosphorylation for ATP synthesis. Knock-down (KD) of the key glycolysis enzyme Phosphofructokinase-2/Fructose-2,6-Bisphosphatase-3 (PFKFB3) impaired tip cell formation by interfering with Notch blockade. Overexpression of PFKB3 overcame the pro-stalk activity of Notch, while treatment with PFKB3 inhibitor, 3-(3-Pyridinyl)-1-(4-Pyridinyl)-2-Propen-1-One (3PO) mimicked the phenotype of PFKB3 KD (De Bock et al., 2013; Schoors et al., 2014; Figure 2B). Recently, a role for the transcription factor Forkhead box O (Foxo1) in endothelial metabolism has also been described. Here, the authors found that Foxo1 is critical in quiescent EC where it would decelerate metabolic activity by reducing glycolysis and mitochondrial respiration via c-Myc. Knock-down (KD) of Foxo1 in EC in mice induced to uncoordinated EC proliferation, leading to vessel hyperplasia (Wilhelm et al., 2016). In a different work, the lactate was also shown to promote angiogenesis through N-Myc Downstream-Regulated Gene 3 Protein (NDRG3) that itself activates the Ras-Erk pathway (Lee et al., 2015). Finally, it was found that hypoxia-mediated VEGF secretion from glioma cells can regulate Glucose Transporter Type 1 (GLUT1) expression in brain endothelium (Yeh et al., 2008). These results show that glucose transport across ECs might be increases by VEGF availability in hypoxic area of tumor and, therefore, promote tumor angiogenesis.

A connection among lipid and glucose metabolism with VEGF secretion was described by Joyal et al. Free Fatty Acid Receptor 1 (Ffar1) reduces GLUT1 expression when free lipids are available. Reduced glucose entry in the VEGF secreting cells causes a decrease of the level of the Krebs cycle intermediate alpha-Ketoglutarate (alpha-KG). Low alpha-KG levels would promote transcription and secretion of VEGF-A (Joyal et al., 2016; Figure 2C). In conclusion, lipid metabolism appears to be vital for availability of VEGFR2 for its ligand, while glucose metabolism is essential for activation of VEGF downstream targets and secretion of VEGF ligand itself. Although promising, these data are far from being completely suitable for treating pathological angiogenesis; until the endothelial autonomous role of these pathways are totally understood.

An emerging concept in angiogenesis is the fact that reactive active species (ROS) and redox events are not just passive events but can actually play a key role during angiogenesis (Panieri and Santoro, 2015). Redox signaling targets various molecules (proteins, lipid, nucleic acid) and occurs in a reversible, specific and dynamic manner (Holmstrom and Finkel, 2014). This balance is regulated by ROS and antioxidants that are in turn produced by specific enzymes. Many angiogenic mechanisms such as VEGFR2 accessibility to its ligand are regulated by ROS directly. The Receptor tyrosine kinase (RTK) domain of VEGFR2 presents two oxidation-sensitive cysteine residues that are kept in a reduced state by antioxidant enzyme Peroxiredoxin-2 (Prx2). Loss of Prx2 increases intracellular level of ROS and oxidation of VEGFR2 on these cysteines, leading to formation of a disulphide bridge. This inactivates VEGFR2 that is no longer able to respond to VEGF (Kang et al., 2011; Figure 2D). Another study suggested that oxidative specie H_2_O_2_ could directly increase VEGFR2 mRNA without affecting VEGFR1 expression (Gonzalez-Pacheco et al., 2006). Phosphorylation of VEGFR3 is regulated by Protein S, that activates Serine Phosphatase SHP2 which de-phosphorylates VEGFR2 leading to its inactivation (Fraineau et al., 2012). Protein S itself is converted into its active form by carboxylation, a process requiring vitamin K as cofactor (Danziger, 2008). As Vitamin K has antioxidant effects, this establish a link between redox signaling and post translational modifications of VEGFR2. Diabetes is a prevalent metabolic disease and most diabetic conditions result in vascular complications due to endothelial dysfunction (Rask-Madsen and King, 2013). ROS generated from hyperglycemia were shown to promote ligand-independent phosphorylation of VEGFR2 and to decrease availability of VEGFR2 at the cell surface (Warren et al., 2014). Interestingly, the reduced cell surface abundance of VEGFR2 can be reversed by treatment with the antioxidant N-acetyl-L-cysteine (NAC), suggesting a causative role for oxidative stress in vascular dysfunction in diabetic conditions (Giacco and Brownlee, 2010).

Reactive oxygen species can also be produced during angiogenesis and have regulatory functions. One of the downstream effectors of VEGFR2 is Ras-Related C3 Botulinum Toxin Substrate 1 (Rac1). Rac1 promotes production of ROS via the NADPH oxidase Nox2 (Diebold et al., 2009; Figure 2D). Interestingly, overexpression of NADPH oxidase Nox4 itself promotes endothelial migration by increasing expression of endothelial Nitric Oxide Synthase (eNOS) (Craige et al., 2011). eNOS is located to the Golgi and has a double function. It can produce Nitric Oxide (NO) when in coupled/ dimer conformation. In absence of cofactors, eNOS shifts to a monomeric form, thus becoming uncoupled and generating ROS such as superoxide anion (•O2^−^) as well as other compounds (Rafikov et al., 2011). Notch is required to maintain NO synthesis by eNOS, as reduction of NO is one the early alteration induced by Notch inhibition (Patenaude et al., 2014; Figure 2E). eNOS coupling and uncoupling is regulated by redox-regulated modifications. In cells with high levels of glutathione disulfide (GSSG), uncoupling of eNOS is promoted by S-Glutathionylation, which modifies two cysteines in the reductase domain of eNOS, leading to ROS accumulation. The antioxidant enzyme Glutaredoxin 1 (Grx1) reverses GSSG-mediated eNOS glutathionylation (Chen et al., 2013; Figure 2E). Recently, a new antioxidant enzyme, Ubiad1, was shown to be critical in ECs. The prenyltransferase Ubiad1 is located in the Golgi and synthesizes CoQ10. Loss of Ubiad1 leads to vascular damage due to ROS accumulation, caused by reduced Coq10 levels and eNOS uncoupling (Mugoni et al., 2013). This work supports a critical role for antioxidants in regulating vascular homeostasis during development and possibly in pathological conditions (Figure 2E).

In addition, links between redox signaling and VEGF secretion were described. VEGF-A promoter presents an oxidative stress response element; and oxidative stress (via H_2_O_2_) induced transcription of VEGF-A, by increasing transactivating activity of Specific protein 1 (Sp1) (Gonzalez-Pacheco et al., 2006). These effects were abolished by addition of the antioxidant NAC (Schafer et al., 2003; Figure 2F). Interestingly, the antioxidant enzyme Manganese-Dependent Superoxide Dismutase (MnSOD) suppresses VEGF-A transcription (Wang et al., 2005; Figure 2F). In conclusion, redox signaling controls angiogenesis by regulating VEGFR2 activity (phosphorylation and oxidation), eNOS coupling and uncoupling; and VEGF-A transcription. Overall, redox events are critical mechanisms in angiogenesis. New reagents and tools are becoming available that will be able to decrypt such critical events at subcellular and tissue levels (Panieri and Santoro, 2015).

## Mechanosensitive cation channels and cilia: new molecular players in flow and mechanical forces-dependent angiogenesis

**Figure 3:**
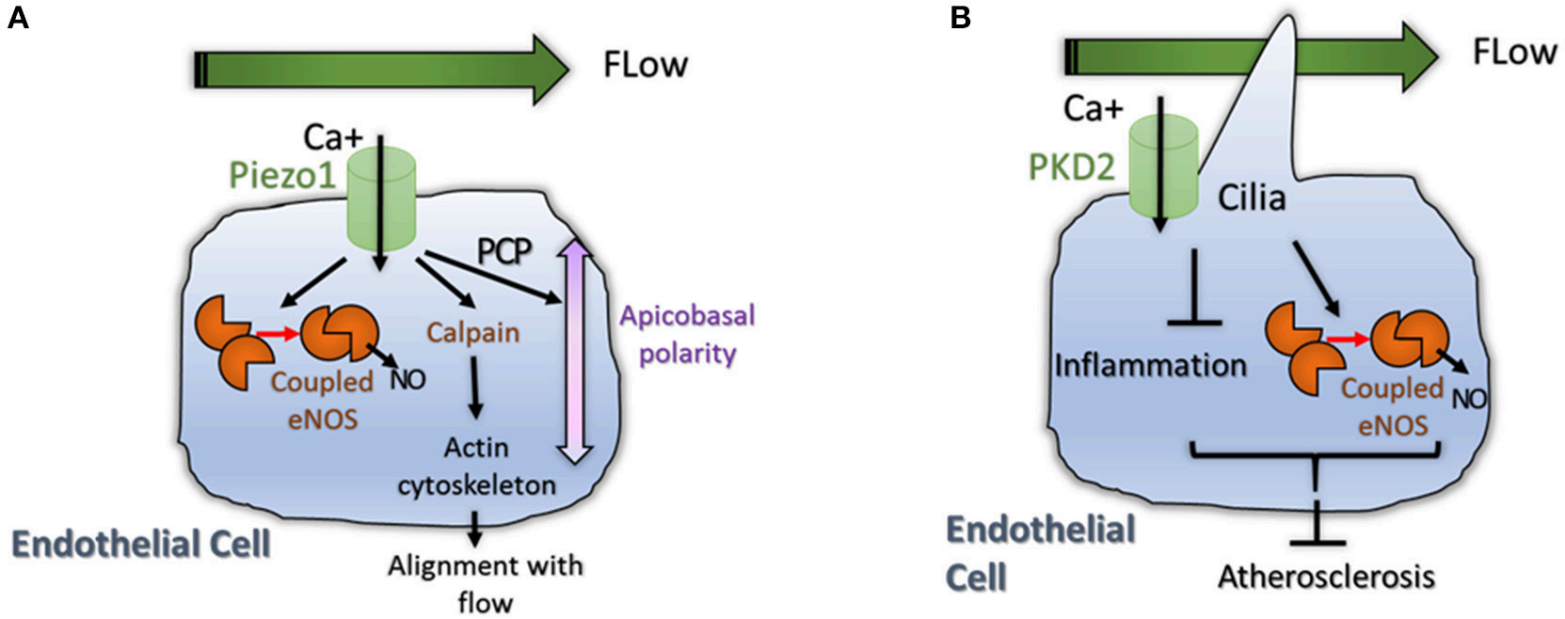
**(A)** Physiological flow induces eNOS signaling and induces reorganization and polarization of the EC. A mechanosensor and calcium transporter called Piezo1 was shown to be activated by flow. High intracellular Ca^+^ levels induce coupling of eNOS but also activate Calpain, an enzyme important for actin cytoskeleton reorganization (Li et al., 2014). High Calcium levels also activate non canonical Wnt pathway (PCP) promoting polarization of the cell; **(B)** Primary Cilia is important for flow sensing in ECs. The cilium is essential for accumulation of intracellular Calcium that promote NO production by eNOS and reduces inflammation (Goetz et al., 2014). Both processes reduce atherosclerosis in aortic endothelium (Dinsmore and Reiter, 2016).

Besides delivering oxygen and nutrients to the tissues, blood flow plays crucial roles in angiogenesis by generating frictional force that develops between flowing blood and the vascular endothelium. ECs covering the inner surface of blood vessels are constantly exposed to different types of shear stress. Shear stress is pulsatile in normal physiology, but can be oscillatory in pathologies such as atherosclerosis, profoundly affecting endothelial function and morphology (Ando and Yamamoto, 2013; Yamamoto and Ando, 2013). It is well-known that shear stress influences the production of NO and Prostacyclin, and may also regulate actin remodeling and signaling pathways such Notch and PCP (Jahnsen et al., 2015). eNOS is a key regulator of NO production that is directly regulated by shear stress through Akt signaling (Peng et al., 2003; Figure 3A).

The mechanism regarding how hemodynamic forces regulate vascular homeostasis is started to be decoded by the identification of the role of Piezo1 in vascular architecture (Figure 3A). Piezo proteins are ion channels mediating mechanosensory transduction acting as sensors of shear stress in EC. Li et al. (2014) showed that endothelial-specific disruption of mouse Piezo1 profoundly disturbed vascular development. Haploinsufficiency was not lethal but displayed abnormalities in mature vessels demonstrating that Piezo1 is determinant for vascular structure in development and adult physiology (Li et al., 2014; Figure 3A). Calcium signaling is also important for signal transduction in ECs submitted to shear stress. For example pulsatile shear stress was shown to induce activation of calcium-sensitive potassium (KCa) channels; the following KCa-dependent hyperpolarization would then trigger eNOS and NO production (Qiu et al., 2001, 2003). This data show that mechanosensitive cation channels might also play pivotal roles in angiogenesis.

Primary cilia are microtubule-based structures present on most mammalian cells that are important for intercellular signaling. Cilia are present on ECs where they project into the flow compartment of a blood vessel. Cilia would act as mechanical sensors of blood flow and modify the response of ECs to biomechanical forces and shear stress (Figure 3B). Two recent papers support a role for cilia in developmental angiogenesis as well as in atherosclerosis. Goetz et al. demonstrate that alterations in ciliogenesis, or expression of the calcium channel PKD2 impair the endothelial calcium level and both perturb vascular morphogenesis (Goetz et al., 2014; Figure 3B). Work in mice, showed that endothelial primary cilia are dispensable for mammalian vascular development but protect against atherosclerosis where shear stress is oscillatory. In atherosclerotic mice models, loss of endothelial cilia increased inflammatory gene expression and decreased eNOS activity, indicating that endothelial cilia inhibit pro-atherosclerotic signaling in the aorta (Dinsmore and Reiter, 2016; Figure 3B). However, more work is crucial to understand the role of cilia during normal and pathological angiogenesis.

## Conclusions and perspective

Due to space limitation we were not able to provide a full coverage of the novel and promising mechanisms important for angiogenesis. However, we have to mention that non-coding RNA-mediated mechanisms are essential in angiogenesis. Recent work from different groups have proposed the role of exosome in regulating angiogenesis via microRNA delivery (Das and Halushka, 2015; Alcayaga-Miranda et al., 2016). MicroRNA-containing exosomes might represent new mediators of intercellular communication among ECs and surrounding tissues (e.g., immune cells, stromal cells) in angiogenesis (Umezu et al., 2014). At the same time, novel reports suggest an expected role for the Metastasis Associated Lung Adenocarcinoma Transcript 1 (MALAT-1) long-non-coding RNAs in regulation of angiogenesis. MALAT-1 is highly expressed in EC. Its silencing regulates the balance from a proliferative to a migratory EC phenotype *in vitro;* while its genetic deletion or pharmacological inhibition reduces vascular growth *in vivo* (Michalik et al., 2014). It was also shown that MALAT1 promotes hypoxia-driven angiogenesis by upregulating pro-angiogenic gene expression in neuroblastoma cells (Tee et al., 2016). Another novel finding is the existence of endothelial progenitors that are promising for regenerative medicine. These cells would be able to regenerate the endothelial network of an existing diseased vascular network. These cells would be highly sensitive to VEGF and have high level of intracellular Ca^2+^, when compared to senescent/ adult endothelial cells (Moccia et al., 2013; Moccia and Poletto, 2015). The functional discovery of novel lncRNAs in cardiovascular disease as well as the function of exosome and endothelial progenitors in endothelial signaling opens the path for novel drug discovery to treat and cure pathological conditions associated to angiogenesis, a notorious but still enigmatic biological process in life.

All these novel findings indicate that we are shifting from a very simple model for angiogenesis, and starting to realize that metabolism, redox signaling, mechanical forces are all crucial for vascular morphogenesis. The next challenges for researchers will be to uncover the functional link among all these conditions in health and diseased conditions. This is even more exciting and intriguing considering that all these different elements are connecting with the pathways classically known to regulate angiogenesis such as VEGF or Notch signaling. Novel technologies, such as redox and metabolic sensors or *in vivo* metabolite tracers are needed to understand these mechanisms spatially and temporally. Integrative and system biology will also be of big help to understand how angiogenic signal networks are regulated in physiology and pathology, dynamically and spatially. We believe that these innovative technologies combined to the novel approaches that have displayed in the papers described in this review, will lead to important advances in the field in the coming years.

## Author contributions

RS and MS draft and write the manuscript. RS did all figures and legends. MS contribute to polish the manuscript.

### Conflict of interest statement

The authors declare that the research was conducted in the absence of any commercial or financial relationships that could be construed as a potential conflict of interest. The reviewer PP declared a shared affiliation with the author MS and states that the process nevertheless met the standards of a fair and objective review.
